# Point-of-care Ultrasound Diagnosis of Emphysematous Cholecystitis

**DOI:** 10.5811/cpcem.2019.11.45337

**Published:** 2020-01-23

**Authors:** Fadwa Al Hammadi, Rasha Buhumaid

**Affiliations:** * Sheikh Khalifa Medical City, Department of Emergency Medicine, Abu Dhabi, United Arab Emirates; † Mohammed Bin Rashid University of Medicine and Health Sciences, Department of Emergency Medicine, Dubai, United Arab Emirates

## Abstract

A 49-year-old male presented to the emergency department with abdominal pain and generalized weakness. The physical examination was positive for right upper quadrant tenderness and positive Murphy’s sign. Point-of-care biliary ultrasound revealed signs of emphysematous cholecystitis. Emphysematous cholecystitis is a rare biliary pathology with a high mortality rate. It differs from acute cholecystitis is many ways. It has unique ultrasound characteristics. This case highlights the use of point-of-care ultrasound to diagnose a rare biliary condition.

## CASE PRESENTATION

A 49-year-old man presented to the emergency department with epigastric abdominal pain. He was known to have multiple myeloma and was on chemotherapy; he also had a mass in the head of the pancreas, which required endoscopic retrograde cholangiopancreatography and stenting one month prior to this presentation. He was hypotensive and tachycardic. Abdominal exam revealed right upper quadrant tenderness and a positive Murphy’s sign. Point-of-care biliary ultrasound revealed gallstones, pericholecystic fluid, and punctate hyperechoic foci in the lumen of the gallbladder (Video). Computed tomography revealed a distended gallbladder with intraluminal gas extending into the inferior surface of the liver (Image). The patient underwent percutaneous cholecystostomy. The bile culture grew the gram-negative bacterium *Prevotella buccae*.

## DISCUSSION

Emphysematous cholecystitis (EC) is diagnosed by the presence of gas in the lumen or the wall of the gallbladder in the setting of acute cholecystitis. It is a rare biliary pathology with a high mortality rate.^1^ EC differs from acute cholecystitis in many ways. It is more common in men and diabetics, and one third of the cases are not associated with cholethiasis.^2^ It is thought to be due to an ischemic event followed by an infection with gas-forming bacteria. The causative organism identified in this case is rare. The most common bacteria associated with this condition are Clostridium species, *Escherichia coli*, Klebsiella species, and anaerobic streptococci.^1^

The appearance of EC on ultrasound differs depending on the amount of gas in the gallbladder. A small amount of gas will produce echogenic foci with reverberation artifact known as ring-down artifact. However, a large amount of gas will produce a band with posterior dirty shadowing.^3^–^4^ Gas can also form multiple echogenic foci that move from the dependent to the independent area within the lumen of the gallbladder, also known as “effervescent gallbladder” or the “champagne” sign.^4^ Computed tomography is more sensitive and specific for the diagnosis of this condition.^5^ EC is a surgical emergency that is managed with intravenous antibiotics and cholecystectomy. Alternatively, percutaneous cholecystostomy is used in patients who are high risk for surgery.^5^

**CPC-EM Capsule**

What do we already know about this clinical entity?

Emergency physicians commonly perform biliary point-of-care ultrasound to identify gallstones and acute cholecystitis.

What is the major impact of the image(s)?

This case describes the use of point-of-care ultrasound to diagnose Emphysematous Cholecystitis (EC), highlighting the unique ultrasound characteristic of this condition.

How might this improve emergency medicine practice?

Using point-of-care ultrasound to diagnose EC (a rare condition with high mortality rate) could help accelerate the diagnosis and management.

## Supplementary Information

Video. Point-of-care biliary ultrasound identifying gallstones (white arrow), pericholecystic fluid (red arrow), and gas in the gallbladder lumen (green arrow).

## Figures and Tables

**Image:**
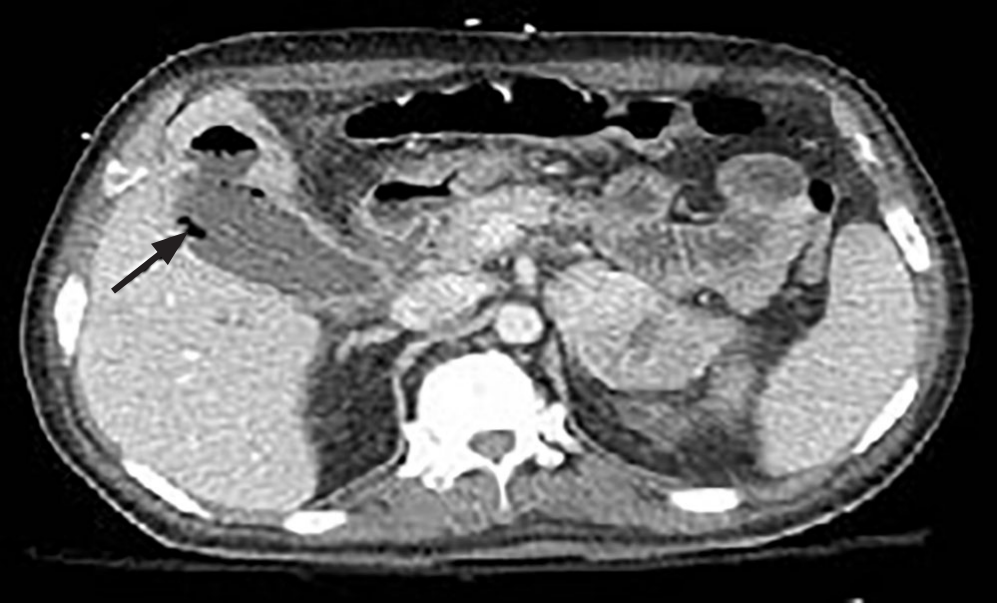
Computerized tomography scan identified distended gallbladder with intraluminal gas (black arrow).
